# Diverging regulatory DNA in adaptive medical AI: US agility and EU accountability in lifecycle governance

**DOI:** 10.3389/fmed.2026.1758708

**Published:** 2026-02-23

**Authors:** Jae Hyun Lee, Boram Choi, Kwunho Jeong, Sang Won Suh, Hwanseok Rhee, Ju Han Kim, Dae-Soon Son

**Affiliations:** 1 Global Research Center, JNPMEDI, Seoul, Republic of Korea; 2 Department of Clinical Research, Onu Institute, Seoul, Republic of Korea; 3 Department of Physiology, Hallym University College of Medicine, Chuncheon, Republic of Korea; 4 Research Institute of Medical-Bio Convergence, Hallym University, Chuncheon, Republic of Korea; 5 Seoul National University Biomedical Informatics (SNUBI), Division of Biomedical Informatics, Seoul National University College of Medicine, Seoul, Republic of Korea; 6 Major in Bio-Healthcare Convergence, Hallym University College of Natural Sciences, Chuncheon, Republic of Korea; 7 Division of Big Data and Artificial Intelligence, Institute of New Frontier Research, Hallym University College of Medicine, Chuncheon, Republic of Korea; 8 Hallym AI-BioHealth R&BD Center, Research Institute of Medical-Bio Convergence, Hallym University, Chuncheon, Republic of Korea

**Keywords:** accountability, adaptive AI, AI Act, lifecycle governance, MDR, PCCP, regulatory DNA, RWE

## Abstract

Medical artificial intelligence (AI) is transitioning from static, rule-based systems into adaptive models capable of continuous learning and iterative refinement. Such adaptivity expands the utility and performance of clinical AI systems across diverse patient populations and real-world conditions. However, these properties challenge regulatory paradigms originally designed for fixed-function medical devices. Although the United States and the European Union share goals of ensuring safety, accountability, and trustworthy performance, their regulatory architectures diverge due to underlying legal-philosophical traditions. The United States employs a common-law, evidence-driven approach centered on the Total Product Life Cycle, using predetermined change-control mechanisms and real-world observational data to support iterative improvement under controlled risk. In contrast, the European Union adopts a civil-law, precautionary model operationalized through the Artificial Intelligence Act, the Medical Device Regulation, and the revised Product Liability Directive, emphasizing ex-ante duties, transparency, traceability, and accountability. Understanding these distinct regulatory DNAs is critical for aligning lifecycle governance of adaptive AI across jurisdictions and ensuring safe, context-responsive innovation.

## 1 Introduction

Medical artificial intelligence is undergoing a shift from locked, rule-based architectures to adaptive systems capable of continuous refinement and contextual responsiveness (1–4). These capabilities are increasingly important as AI models encounter heterogeneous patient populations, evolving clinical knowledge, and variable real-world environments (5, 6).

In this Perspective, we use “adaptive medical AI” to encompass systems whose performance or behavior may change after deployment through ongoing learning, recalibration, or scheduled updates. Importantly, adaptation is not a single technical mode: it may occur through continuous learning mechanisms that update model parameters in near real time, or through periodic updates (e.g., batch retraining or recalibration) released under controlled governance. These distinctions are significant from a regulatory perspective because the frequency and scope of change shape risk, oversight feasibility, and lifecycle evidence needs.

Yet legacy medical-device regulation—built around linear sequences of manufacture, approval, and tightly constrained post-market modification—was never designed for systems that evolve after deployment (7, 8). These frameworks assume that safety and effectiveness can be validated at a fixed moment and that system performance remains stable during commercial use. Adaptive AI challenges each of these premises.

Concrete tensions have already emerged in practice. Developers may struggle to determine when routine recalibration constitutes “maintenance” versus a regulated modification that could require additional review, while regulators face uncertainty in assessing safety and effectiveness when real-world performance diverges from its originally validated state. Such frictions are amplified by well-described phenomena such as calibration drift in clinical prediction models, which can occur even without structural retraining and may necessitate periodic recalibration to maintain clinical reliability (4).

Accordingly, governing adaptive medical AI requires more than extending legacy device paradigms: it demands explicit definitions of permissible update types, pre-specified validation and documentation expectations, and post-market monitoring triggers that determine when performance changes warrant escalation, notification, or re-assessment.

This Perspective examines how the United States (US) and the European Union (EU)—despite shared commitments to safety, accountability, and trustworthy innovation—have developed fundamentally different regulatory identities rooted in US common-law incrementalism and EU civil-law codification (9, 10). Understanding these regulatory DNAs is essential for shaping future governance of adaptive AI systems (Table 1).

**Table 1:** Key regulatory contrasts between US and EU approaches to lifecycle governance of adaptive medical AI.

Dimension	United States (FDA)	European Union (AI Act + MDR + PLD)
Regulatory posture	Evidence-driven, iterative oversight across the TPLC	Precautionary, ex-ante duties emphasizing predictability and rights
Primary lifecycle tool for updates	PCCP enabling pre-authorized, bounded changes	Conformity assessment-centered control of change; limited pre-authorization for adaptation
Role of real-world data	RWE as a feedback loop to monitor drift and validate updates	Data as compliance evidence and documentation; post-market signals via MDR surveillance/vigilance
Transparency and documentation	Strong emphasis for PCCP submissions; may vary by implementation capacity	Broad statutory duties for documentation, traceability, and governance
Accountability and liability logic	Case-by-case liability through tort-based adjudication	Structural accountability with statutory duties and liability presumptions (PLD)
Innovation friction	Lower pre-market friction; higher reliance on post-market monitoring capacity	Higher pre-market friction; sandboxes provide guided pathways without waiving duties

In the sections that follow, we unpack how these distinct regulatory DNAs manifest in the US and the EU, tracing the legal traditions, oversight tools, and lifecycle mechanisms that structure their approaches. We then analyze the divergent implications these choices create for innovation, safety, accountability, and the prospects for global harmonization.

## 2 Diverging approaches to adaptive medical AI

### 2.1 The US approach: evidence-driven agility (common-law logic)

The US approach to adaptive medical AI tends to treat model evolution not as an exception but as an anticipated feature of real-world deployment. This stance aligns with the pragmatism of the US common-law tradition, which often addresses uncertainty through incremental adjustment rather than comprehensive ex-ante control. Within this context, the Total Product Life Cycle (TPLC) framework provides a conceptual foundation for US oversight. The FDA has further advanced this lifecycle view through initiatives such as the Total Product Life Cycle Advisory Program (TAP) Pilot, which seeks to support earlier and more continuous engagement across the lifecycle for eligible technologies (11). Rather than viewing medical AI as a fixed-function device validated at a single moment in time, a TPLC orientation assumes that performance can shift as models encounter heterogeneous patient populations, new clinical knowledge, and changing real-world environments (11). In principle, this lifecycle framing supports continued regulatory engagement across development, deployment, and post-market monitoring, reflecting the premise that safety for adaptive systems may depend on ongoing oversight rather than a single front-loaded assurance event.

This lifecycle foundation is operationalized through Predetermined Change Control Plans (PCCPs), which translate a dynamic view of AI into a structured administrative tool. In the FDA’s current guidance, a PCCP is expected to describe (i) the planned device modifications, (ii) the associated methodology to develop, validate, and implement those modifications, and (iii) an assessment of the impact of those modifications (12). Reviewed as part of a marketing submission, a PCCP functions as a regulatory pre-commitment: developers can implement pre-specified, bounded updates without submitting a new marketing application for each change, while regulators maintain predictability through upfront specification of update methods, evidence plans, and control limits (12).

Yet adaptability alone does not guarantee safety. To close this loop, the US system relies heavily on Real-World Evidence (RWE) as an empirical feedback mechanism. RWE enables regulators and developers to evaluate whether updates implemented under a PCCP maintain acceptable performance when exposed to real clinical variation, but its value depends on whether the underlying real-world data are fit-for-purpose (e.g., relevant, reliable, and sufficiently traceable for the regulatory question) (13). Rather than relying exclusively on controlled trials—which are often infeasible for frequently updated systems—RWE programs can be used to monitor drift, heterogeneity effects, and calibration changes *in situ*, ideally under a monitoring plan that pre-defines the target metrics, drift thresholds, reporting cadence, and corrective actions. For instance, calibration drift has been documented in clinical prediction models such as those used for acute kidney injury risk estimation, highlighting the need for ongoing monitoring and recalibration in practice (4). Taken together, TPLC provides the conceptual structure, PCCPs institutionalize planned adaptation, and RWE supplies the evidence needed to validate ongoing change.

However, evidence-driven agility is not operationally frictionless. PCCP- and RWE-enabled iteration presupposes sustained access to high-quality and representative real-world data—an assumption that may not hold uniformly across institutions, regions, or patient subgroups due to interoperability barriers, privacy constraints, and fragmented data stewardship. Continuous performance monitoring also imposes nontrivial technical and organizational burdens, including data governance, drift-detection infrastructure, subgroup performance auditing, and clear escalation pathways when degradation is detected. Critically, these burdens are often shared across developers and healthcare institutions, meaning that the practical effectiveness of the US approach depends on unevenly distributed capacities and may perform best in well-resourced settings.

### 2.2 The EU approach: precautionary accountability (civil-law logic)

In contrast, the EU adopts an orientation rooted in civil-law codification, fundamental rights protection, and the precautionary principle. Rather than assuming that post-deployment adaptation can be managed primarily through iterative oversight, the EU regulatory DNA emphasizes ex-ante duties designed to minimize uncertainty before market entry. These duties are distributed across overlapping instruments. Under the AI Act, many medical AI systems are categorized as high-risk, triggering requirements for risk management, data governance, technical documentation, logging and traceability, human oversight, and demonstrated accuracy and robustness (14). In parallel, when medical AI is placed on the market as a medical device software function, the MDR imposes conformity assessment, clinical evaluation, and quality-management obligations that structure evidence generation and control of change (15). Recent EU guidance (MDCG 2025–6) explicitly frames these regimes as simultaneously and complementarily applicable for medical-device AI and encourages integrated compliance approaches rather than parallel, duplicative systems (16). The interplay between these instruments can create both redundancy (e.g., documentation and governance) and complementarity (e.g., medical-device lifecycle controls supporting AI-specific duties), reinforcing the EU’s precautionary posture (16).

Importantly, the EU framework is not purely static. The MDR embeds lifecycle governance through post-market surveillance and vigilance obligations, requiring manufacturers to collect and evaluate experience gained from devices in use and to respond to incidents and performance concerns (15). For adaptive or frequently updated software, these post-market duties can become a key channel through which real-world performance signals are detected and acted upon, even when the regulatory pathway for pre-authorized adaptation remains comparatively constrained.

This ex-ante posture is reinforced by the Revised Product Liability Directive (PLD), which modernizes liability law to address the unique risks of adaptive and opaque AI systems alongside the AI Act and MDR. The PLD introduces expanded duties to update, clearer obligations around transparency, and presumptions of defect when manufacturers cannot demonstrate adequate documentation or post-market diligence (17). These provisions shift evidentiary burdens toward developers, embedding accountability structurally within statutory duties rather than relying primarily on case-by-case adjudication.

Recognizing, however, that strict ex-ante requirements can impede innovation, the EU has introduced Regulatory Sandboxes as a controlled mechanism to support experimentation without undermining its precautionary architecture (14). Participation in a sandbox primarily helps developers interpret and operationalize compliance expectations (e.g., documentation, data governance, and testing plans) through close regulatory accompaniment. Sandboxes therefore aim to reduce regulatory uncertainty and improve readiness for conformity assessment, rather than to waive substantive obligations or substitute for post-market lifecycle duties.

### 2.3 Divergent implications for innovation, safety, and trust

These regulatory DNAs—evidence-driven agility in the US and precautionary accountability in the EU—do more than create procedural differences. They generate distinct trajectories for innovation, risk distribution, and public trust. Understanding these implications is essential for navigating global deployment of adaptive medical AI.

A key divergence concerns the trade-off between timeliness and predictability. The US model accelerates innovation by allowing models to evolve under PCCPs and validating performance through RWE. This responsiveness can yield rapid benefits but also concentrates risk in the post-market phase if drift emerges undetected. Conversely, the EU’s ex-ante gatekeeping fosters predictability and public trust through rigorous documentation and conformity assessment but slows adaptation, increasing the risk of outdated models persisting in clinical use.

A second divergence appears in the logic of data. In the US, data primarily functions as fuel for iteration—a resource used to fine-tune, recalibrate, and enhance models throughout their lifecycle. In the EU, data functions as evidence for compliance, shaping developer incentives toward robust governance, traceability, and documentation infrastructures.

A third divergence involves allocation of responsibility. The US tort system evaluates liability case by case, enabling contextual flexibility but generating uncertainty about ultimate accountability. In contrast, the EU employs structural accountability, where statutory duties and presumptions of defect place predictable responsibility on developers, enhancing trust but increasing regulatory burden.

Taken together, these differences produce distinct innovation ecosystems. The US model rewards rapid experimentation under managed uncertainty, while the EU model prioritizes reliability and fundamental-rights safeguards through stronger ex-ante predictability. Neither approach is inherently superior; each reflects a coherent value hierarchy encoded in its regulatory DNA.

## 3 Discussion

The divergent regulatory DNAs of the US and the EU shape not only how adaptive AI systems evolve after deployment but also how developers, clinicians, and regulators interpret their ongoing responsibilities within the broader innovation ecosystem. The US approach enables iterative refinement through PCCPs and RWE (11–13, 18), supporting context-responsive improvements that help maintain clinical relevance in dynamic environments. This agility is particularly advantageous when rapid recalibration is required to prevent model drift or performance degradation, especially in clinical contexts where population characteristics shift or emerging evidence demands timely updates. In contrast, the EU’s documentation-rich and rights-centered framework strengthens transparency, explainability, and legal accountability—features essential for public trust and system legitimacy (14, 15, 17, 19–21). These attributes are especially valuable for population-level deployments that require predictability, traceability, and clearly allocated responsibility throughout the lifecycle, ensuring that changes do not undermine established safeguards (22).

These approaches reflect partially competing priorities: rapid clinical responsiveness through iterative updating versus predictable accountability anchored in ex-ante assurance. A workable “happy medium” therefore requires risk-based co-lifecycle governance that permits bounded adaptation where benefits are clear and risks are manageable, while reserving stricter reassessment for changes that could materially alter intended use, clinical impact, or safety profiles. Achieving this balance also depends on coordinated roles among key stakeholders—including regulators, notified bodies (in the EU), developers, healthcare institutions, clinicians, and patients—because monitoring, documentation, and escalation cannot be executed by a single actor alone. Operationally, developers must maintain update documentation and performance monitoring plans, healthcare institutions must enable data capture and incident reporting in routine workflows, and regulators/notified bodies must define acceptance criteria and escalation pathways that translate post-market signals into governance actions.

The complementary strengths of the two systems create opportunities for meaningful cross-jurisdictional learning. PCCP-based update structures in the US may inform future EU mechanisms for managing permissible model evolution without compromising ex-ante assurance, offering a pathway for integrating controlled adaptivity into the EU’s traditionally static conformity assessment processes. Conversely, EU data-governance duties and liability presumptions can enrich US expectations for transparency, auditability, and post-deployment obligation, highlighting areas where US oversight may benefit from more formalized assurances. As adaptive AI increasingly intersects with cross-border clinical workflows, distributed datasets, and multinational deployment pipelines, alignment between these regulatory DNAs becomes essential for ensuring practical interoperability, reducing regulatory fragmentation, and sustaining safety across jurisdictions.

In our view, future global harmonization efforts must explicitly prioritize co-lifecycle governance that couples US-style PCCP-driven adaptability with EU-derived accountability and documentation safeguards. Neither agility nor precaution alone is sufficient for governing adaptive medical AI; instead, durable trust will require a hybrid model that institutionalizes both empirical feedback loops and rights-based protections. Such an approach acknowledges the inevitability of model evolution while ensuring that changes remain transparent, auditable, and aligned with established ethical and legal expectations (23).

Global Harmonization Implications. Meaningful convergence between the US and EU will require operational mechanisms rather than conceptual alignment alone. We propose three actionable components:

(1) Shared taxonomy of permissible software changes (e.g., calibration-only updates, retraining within a fixed architecture, and architecture-level changes), linked to clear regulatory triggers, validation expectations, and notification thresholds—thereby enabling pre-authorized, bounded updates without defaulting to full return-to-gate assessment.
(2) Standardized documentation and traceability packages for each update (e.g., change logs, data provenance, subgroup performance reports, and human-oversight provisions), improving auditability and clarifying accountability when performance shifts in real-world use.
(3) Interoperable expectations for post-market performance monitoring, including minimum drift-detection metrics, reporting cadence, and escalation pathways when degradation is detected, to reduce fragmentation for multinational deployment and to avoid divergent model behavior across markets.

In parallel, structured early dialogue mechanisms—such as coordinated scientific advice or sandbox-based accompaniment—could help developers design evidence plans that satisfy both PCCP-style adaptability and EU-grade accountability from the outset.

Together, these steps would operationalize co-lifecycle governance by combining planned iteration, empirical feedback, documentation, and clearly allocated responsibility. Such integration supports safe, transparent, and context-responsive innovation across global health systems and provides a foundation for multinational trust in adaptive AI. As the clinical, legal, and technical stakes of adaptive models continue to rise, aligning the agility of the US system with the accountability of the EU will be critical for shaping a globally coherent and ethically robust regulatory future. Societally, such co-lifecycle governance can improve timely access to safer and more current models while sustaining public trust through transparency and clear responsibility; however, it may also amplify inequities if monitoring and documentation costs fall disproportionately on resource-limited settings, underscoring the need for shared infrastructure and proportional requirements.

## Data Availability

The original contributions presented in the study are included in the article/supplementary material, further inquiries can be directed to the corresponding author.
